# Choroidal structure investigated by choroidal vascularity index in patients with inherited retinal diseases

**DOI:** 10.1186/s40942-023-00457-w

**Published:** 2023-03-28

**Authors:** Kia Bayat, Kiana Hassanpour, Hamideh Sabbaghi, Sahba Fekri, Narsis Daftarian, Tahmineh Motevasseli, Fatemeh Suri, Bahareh Kheiri, Mehdi Yaseri, Hamid Ahmadieh

**Affiliations:** 1 Ophthalmic Research Center, Research Institute for Ophthalmology and Vision Science, Shahid Beheshti University of Medical Sciences, Tehran, Iran; 2 School of Medicine, Shahid Beheshti University of Medical Sciences, Tehran, Iran; 3 Department of Ophthalmology, Labbafinejad Medical Center, Shahid Beheshti University of Medical Sciences, Tehran, Iran; 4 Ophthalmic Epidemiology Research Center, Research Institute for Ophthalmology and Vision Science, Shahid Beheshti University of Medical Sciences, Tehran, Iran; 5 Ocular Tissue Engineering Research Center, Research Institute for Ophthalmology and Vision Science, Shahid Beheshti University of Medical Sciences, Tehran, Iran; 6 Department of Epidemiology and Biostatistics, Tehran University of Medical Sciences, Tehran, Iran

**Keywords:** Choroidal vascularity index, Inherited retinal dystrophies, Choroid, Optical coherence tomography

## Abstract

**Purpose:**

To evaluate the choroidal structure in patients with inherited retinal diseases (IRDs) by investigating the choroidal vascularity index (CVI).

**Methods:**

The present study was conducted on 113 IRD patients and 113 sex- and age-matched healthy individuals. Patients’ data was extracted from the Iranian National Registry for IRDs (IRDReg®). Total choroidal area (TCA) was determined between retinal pigment epithelium and choroid-scleral junction,1500 microns on either side of the fovea. Luminal area (LA) was considered as the black area corresponding to the choroidal vascular spaces, following Niblack binarization. CVI was calculated as the ratio of the LA to the TCA. CVI and other parameters were compared among different types of IRD and the control group.

**Results:**

The IRD diagnosis included retinitis pigmentosa (n = 69), cone-rod dystrophy (n = 15), Usher syndrome (n = 15), Leber congenital amaurosis (n = 9), and Stargardt disease (n = 5). Sixty-one (54.0%) individuals of each of the study and control groups were male. The average CVI was 0.65 ± 0.06 in the IRD patients and 0.70 ± 0.06 in the control group (P < 0.001). Accordingly, the average of TCA and LA were 2.32 ± 0.63 and 1.52 ± 0.44 mm [1] in patients with IRDs, respectively. The measurements for the TCA and the LA were significantly lower in all subtypes of IRD (P-values < 0.05).

**Conclusion:**

CVI is significantly lower in patients with IRD than in healthy age-matched individuals. Choroidal changes in IRDs may be related to the changes in the lumen of the choroidal vessels rather than the stromal changes.

**Supplementary Information:**

The online version contains supplementary material available at 10.1186/s40942-023-00457-w.

## Introduction

Inherited retinal diseases (IRDs) are a heterogeneous group of retinal disorders associated with progressive deterioration of the photoreceptors’ function. ^1^With the prevalence of 1 in every 3,000 individuals, IRDs are the most prevalent hereditary cause of severe visual impairment in children and the working-age population, which may progress eventually to irreversible complete sight loss [1–3].

Findings in clinical settings have been proposed for the diagnosis of IRDs. However, there is an overlap of common signs and symptoms such as severely decreased central vision, visual field defect, nyctalopia, nystagmus, and even the presence of normal-looking fundus among multiple subtypes of IRDs. Therefore, clinical findings are not a reliable method for the precise identification of different subtypes [2, 4]. Electrophysiological testing and multiple retinal imaging modalities such as optical coherence tomography (OCT), color fundus photography, and autofluorescence imaging (AF) have been acknowledged as proper techniques for detecting IRDs [2, 5–7].

Even though IRDs basically involve the dysfunction of photoreceptors and retinal pigment epithelium (RPE), recent studies have reported alterations of choroidal structure in these diseases [8–11]. Hence, quantitative assessment of both vascular and stromal components of the choroidal structure can be a notable tool for diagnosing IRDs and distinguishing different subtypes of these retinal disorders. Choroidal thickness has been considered a substantial marker for detecting changes in choroidal structure on OCT images of IRD patients. Recent studies have discovered thinning of choroid in IRD cases [12–14]. EDI-OCT however, in reporting choroidal consistency, is incapable of discriminating between stromal versus vascular changes of choroidal structure [15–19].

Choroidal vascularity index (CVI) is a novel OCT-based index which is defined as the proportion of vascular areas to that of the total choroidal area. It has already been employed to investigate choroidal changes in retinal vein occlusion [20], diabetic retinopathy [21] and age-related macular degeneration (AMD) [22]. In this study, we aimed to employ CVI for detecting choroidal changes in retinal dystrophies and also for distinguishing different subtypes of IRDs.

## Methods

​​In this cross-sectional study, the clinical and imaging data of the right eyes of 113 patients with IRDs were extracted from the Iranian National Registry for IRDs (IRDReg®) [22]. In addition, 113 sex- and age-matched healthy individuals were enrolled in the study as the control group.

Written informed consent was obtained from all subjects. All study procedures adhered to the tenets of the Declaration of Helsinki. The study was approved by the Ethics Committee at the Ophthalmic Research Center, Research Institute for Ophthalmology and Vision Science with the code number: IR.SBMU.ORC.REC.1396.15.

Patients were examined at Labbafinejad Medical Center, a tertiary referral center in Tehran, Iran. Diagnosis of IRD variations was made by a board-certified retina specialist after evaluation of clinical findings and electroretinogram (ERGs) results. Patients underwent genetic testing to confirm the diagnosis [23].

Demographic data and baseline clinical information including the present age and the age at disease onset, gender, past medical history and visual symptoms such as visual field defects and decreased central vision were recorded. All individuals underwent a complete ophthalmologic examination including best-corrected visual acuity (BCVA) measured by the Snellen chart, color vision evaluated by Ishihara pseudoisochromatic 38-plates, slit-lamp examination of the anterior segment, intraocular pressure (IOP) measured by a Goldmann applanation tonometer, dilated fundus examination performed by + 78 diopter (D)/+90 D lenses and indirect ophthalmoscopy.

Exclusion criteria for this study were severe cystoid macular edema, history of intraocular surgery, presence of other retinal diseases such as diabetic retinopathy and AMD, mature cataract, glaucoma and myopic refractive errors greater than − 6.0 diopters. Patients with a confirmed diagnosis of systemic hypertension or diabetes were also excluded. Images with poor quality and images with unidentifiable center of the macula were excluded as well.

Choroidal images were provided by the EDI-OCT (Heidelberg Spectralis System, Heidelberg Engineering, Heidelberg, Germany). To minimize the effect of diurnal variations of choroidal structure, EDI-OCT images were obtained from 15:00 to 17:00. EDI-OCT images were analyzed by ImageJ software (version 1.53; National Institutes of Health, USA; http://imagej.nih.gov/ij/).

Processing of images was carried out by adopting the protocol reported by Sonoda et al [24]. The total choroidal area (TCA) was determined by the “Polygon Selection” tool as the area between the basal margin of the RPE and the choroid-scleral junction with a width of 1500 μm toward the temporal and nasal sides each, where the fovea was at the center. For measurement of the choroidal luminal area (LA), three choroidal vessels with lumens larger than 100 μm were selected by the “Oval Selection” tool. To keep the image noise at the least possible level, the average brightness of the luminal areas chosen in the previous step was calculated by the “Measure” tool and subsequently was set as the minimum value of the image reflectivity in the “Brightness/Contrast” tool.

**Fig. 1:**
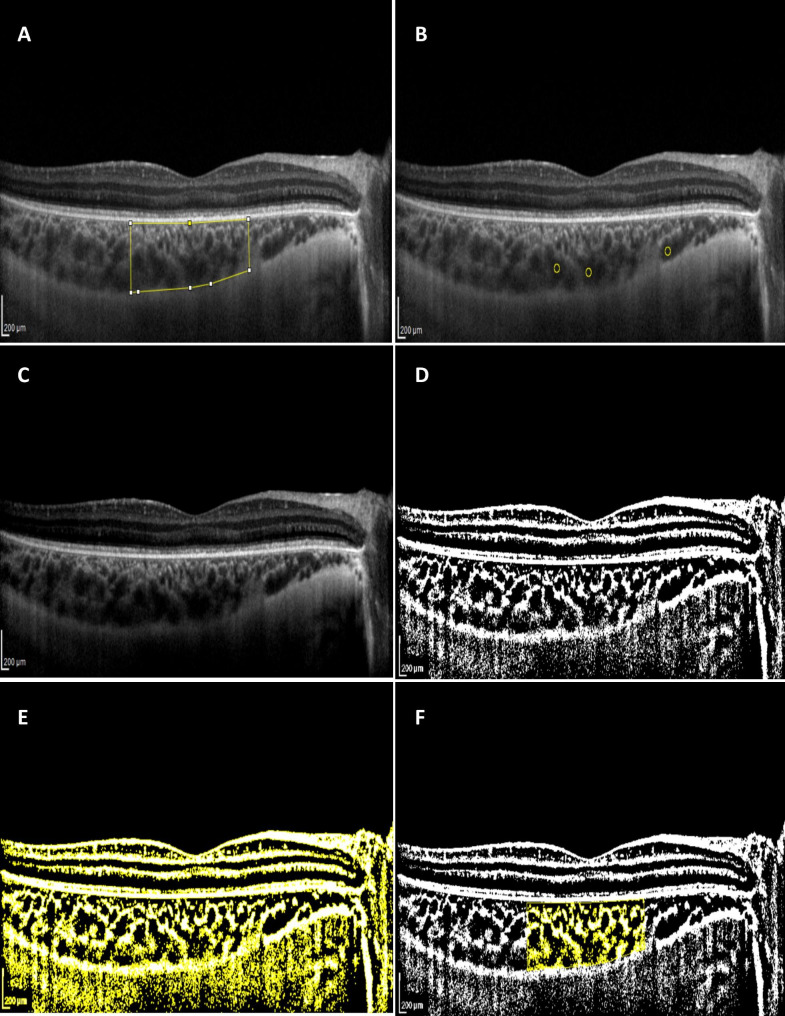
Enhanced depth imaging (EDI) optical coherence tomography images of a patient with RP. The light areas in choroid are the stromal areas and the dark areas are the luminal areas **A**. the total choroidal area (TCA) was determined as the area between RPE and the choroid-sclera junction in the subfoveal choroid. The examined area was set to be 3000 μm wide **B**, **C**. the average brightness of the three choroidal vessels with lumens larger than 100 μm was set as the minimum value of the image reflectivity in brightness/contrast tool **D**. the image was converted to a binary image using Niblack auto local threshold tool **E**, **F**. color threshold tool was applied to specify luminal areas from stromal areas

To make the binarization process possible, the type of image was downgraded to an 8-bit image. Binarization was then performed using the Niblack method of the “Auto Local Threshold” tool. The binarized image was converted back to a red green blue (RGB) image; a “color threshold tool” was applied to specify luminal areas or dark pixels from the stromal areas or light pixels. LA was measured by the “analyze-measure tool”. CVI was calculated as the ratio of LA to TCA, and the stromal area (SA) was computed by the subtraction of LA from TCA (Fig. 1).

### Statistical analysis

To describe the data, mean, frequency and standard deviation, median and interquartile range were used. Choroidal parameters were compared between cases and controls by Mann-Whitney test. The differences among IRDs groups and controls were investigated by Kruskal-Wallis test, then comparisons between levels of IRDs groups and controls were performed. In this evaluation, multiple comparisons were considered by Bonferroni method. Although the groups were matched for age and sex, we adjusted the effect of age and sex for possible residual confounding effects by general linear model. Correlation analysis was performed using Spearman correlation coefficient. SPSS (IBM Corp. Released 2019. IBM SPSS Statistics for Windows, Version 26.0. Armonk, NY: IBM Corp) was used to analyze the data. P-value less than 0 < 05 was considered statistically significant.

## Results

A total of 113 patients with IRD, including 69 with retinitis pigmentosa (RP), 15 with cone-rod dystrophy (CRD), 15 with Usher syndrome, 9 with Leber congenital amaurosis (LCA) and 5 with Stargardt disease (STGD) were recruited. In addition, 113 healthy individuals were enrolled. Sixty-one (54.0%) individuals were male in each of the IRD and control groups (Table 1). The patients were very genetically heterogenous and we did not find any association between the causative genes and the choroidal structure (data not shown).

**Table 1:** Demographic characteristics of study groups

	Groups		
	Control	IRDs	P-Value
Number of subjects	113	113	
Age in years			
≤ 20	4	9	
21–40	47	54	
41–60	33	43	
61–80	4	7	
Overall (Mean ± SD)	36.89 ± 11.87	38.55 ± 13.62	0.72*
Gender			
Female [No. (%)]	52 (46.0)	52 (46.0)	1**
Male [No. (%)]	61 (54.0)	61 (54.0)	

IRD: Inherited retinal disease; *Based on Mann-whitney; ** Based on chi-square

The average CVI was 0.65 ± 0.06 in the IRD patients and 0.70 ± 0.06 in the control group (P < 0.001). Accordingly, the average of TCA and LA was 2.32 ± 0.63 mm^2^ and 1.52 ± 0.44 mm [1] in patients with IRDs, respectively. The corresponding values for the control group were 2.68 ± 0.6 mm^2^ and 1.85 ± 0.36 mm [1]. In the univariate analysis, the TCA and LA measurements were significantly lower in the IRD patients as compared with the control group (all Ps < 0.001); however, there was no statistically significant difference in terms of the stromal area (SA) between cases and controls (0.8 ± 0.24 mm [1] vs. 0.83 ± 0.3 mm [1], P = 0.47).

Multivariable analysis revealed statistically significant difference between cases and controls in terms of all of the indexes, including TCA, LA, CVI (P-values < 0.001, < 0.001, and < 0.001, respectively) after adjusting for age and sex (Table 2).

**Table 2:** Comparison of choroidal parameters among IRD groups and healthy controls

	RP		STGD		Cone- Rod Dystrophy		LCA		Usher Syndrome		Control		P-value*
Total Choroidal Area	2.24 ± 0.53		2.23 ± 0.35		2.29 ± 0.74		2.53 ± 0.97		2.62 ± 0.61		2.49 ± 0.5		< 0.001
Statistically significant different groups (P-value)**	Control (< 0.001)										RP (< 0.001)		
Luminal Area	1.46 ± 0.38		1.44 ± 0.24		1.43 ± 0.5		1.57 ± 0.62		1.82 ± 0.46		1.78 ± 0.35		< 0.001
Statistically significant different groups (P-value)**	Control (< 0.001) Usher Syndrome (P = 0.045)				Control (0.015)				Usher Syndrome (P = 0.045)		RP (< 0.001) CRD (0.015)		
CVI	0.65 ± 0.06		0.65 ± 0.06		0.62 ± 0.04		0.62 ± 0.04		0.69 ± 0.04		0.72 ± 0.04		< 0.001
Statistically significant different groups (P-value)**	Control (< 0.001)				Control (< 0.001) Usher (0.005)		Control (0.003) Usher (0.045)		CRD (0.005) LCA (0.045)		RP (< 0.001) CRD (< 0.001) LCA (0.003)		
Stromal Area	0.77 ± 0.22		0.79 ± 0.21		0.86 ± 0.25		0.96 ± 0.39		0.8 ± 0.2		0.7 ± 0.2		0.77

IRD: Inherited Retinal Disease; RP: Retinitis Pigmentosa; STGD: Stargardt Disease; LCA: Leber Congenital Amaurosis; CVI: Choroidal Vascularity Index; CRD: Cone-rod dystrophy; * Based on general linear model; ** multiple comparisons considered by Bonferroni method. Adjusted P values have been reported

### Correlation analysis

Regarding the correlation of age and indexes, TCA, LA, and SA correlated with age (P = 0.003, 0.004, and 0.018, respectively; Spearman correlation coefficients were − 0.215, -0.232 and − 0.138). (Table 3) However, CVI and age were not correlated (Spearman correlation coefficient and P-value were − 0.058 and 0.531, respectively).

**Table 3:** Correlation between choroidal parameters and age

	TCA	LA	CVI	SA	Total Thickness
Spearman Correlation	-0.215	-0.232	-0.045	-0.138	-0.433
P-value	0.002	0.001	0.53	0.051	0.001

TCA: Total choroidal area, LA: Luminal area; CVI: choroidal vascularity index; SA: Stromal area

### Comparisons among different IRDs

**Fig. 2:**
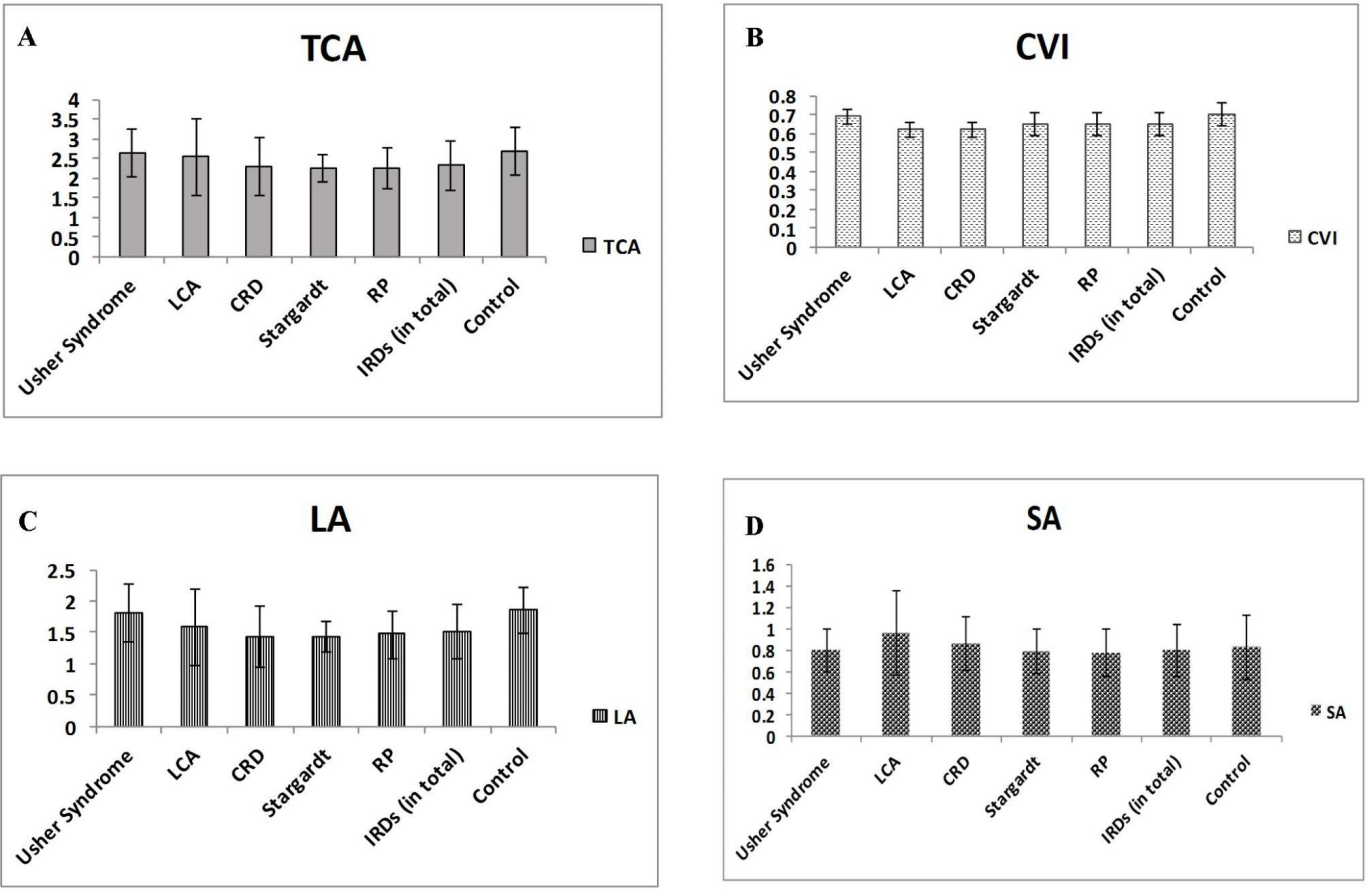
Total choroidal stroma (TCA), Choroidal Vascularity Index (CVI), Luminal Area (LA), and Stromal Area (SA) are demonstrated in different subtypes of inherited retinal dystrophies. (**A-D**)

There were statistically significant differences between RP vs. controls, CRD vs. Usher syndrome, CRD vs. controls, LCA vs. Usher syndrome, and LCA vs. controls in terms of the CVI. (Adjusted P-values: <0.001, 0.005, < 0.001, 0.045, and 0.003, respectively). There was a significant difference between RP vs. controls regarding TCA (P < 0.001). There was also a significant difference between RP vs. controls (P < 0.001), and CRD vs. controls in terms of LA (P = 0.015). There was no significant difference between subgroups of IRDs and healthy individuals regarding SA (Table 2; Fig. 2). CVI was comparable between RP and STGD (P: >0.99).

## Discussion

In this study, univariate and multivariable analysis indicated lower amounts of CVI, TCA, and LA in patients with IRDs than in the age- and sex-matched controls. The univariate analysis did not reveal a statistically significant difference between cases and controls regarding SA. However, patients with IRDs had a significantly lower SA than healthy individuals after adjusting for age and sex. We also found a correlation between age and three studied indices, including TCA, LA, and SA. Reduced amounts of all three indices were seen with increasing age. Our analysis did not reveal a significant correlation between age and CVI. To the best of our knowledge, the present study is the largest one investigating CVI in patients with genetically confirmed diagnosis of IRD.

Comparing CVI between IRD subgroups and controls, RP, LCA, and CRD had significantly lower values compared with the control group. There was also a significant difference between Usher and LCA and also between Usher and CRD. CVI in Usher was significantly greater than LCA and CRD.

Our results correlate with previous studies investigating CVI in patients with IRDs. RP followed by STGD are the most studied dystrophies in which CVI consistently shows reduction [8, 25, 26]. Wei and associates [8] investigated CVI in 17 patients with RP, four patients with STGD, and three patients with CRD as compared to healthy controls. The authors noticed lower mean CVI in IRDs than in controls. Our results are consistent with Wei et al’ s study, while there is a larger sample size in each subgroup in the current study. Ratra and colleagues investigated CVI in 39 patients with STGD as compared to the healthy controls. CVI was significantly decreased in patients with STGD. The authors concluded that CVI is a more robust tool than the subfoveal choroidal thickness (SFCT) measurement to evaluate the choroidal structure [25]. In the present study, CVI was lower in patients with STGD as compared with controls; however, this difference did not reach statistical significance possibly due to the small sample size of the STGD group.

There are several mechanisms explaining the reduced CVI in patients with IRD. First, the choroidal vessels are responsible for the blood supply of RPE and outer retina. In most IRDs, the primary pathology comprises atrophy of the RPE and the outer retina. The reduction in choroidal vasculature could be viewed as a primary pathology or an autoregulatory consequence of RPE and outer retina attenuation. This needs to be evaluated in future studies.

The luminal changes were more prominent than the stromal changes in IRD patients. This finding correlates with previous studies investigating choroidal vasculature in these patients. Despite stability of the stromal area, the changes in the lumen were prominent. This finding supports the possible changes that occur in choroidal vessels in patients with IRDs.

Changes in choroid are well-documented in patients with RP. Choroidal thinning has been shown in histopathological studies and OCT [14]. Additionally, magnetic resonance imaging, Doppler, and laser speckle flowgraphy have demonstrated lower choroidal blood flow in RP patients [27].

Comparing the subgroups of the IRDs, CVI was reduced in RP, CRD, LCA, and Usher when also compared to the healthy controls. Of note, CVI was comparable between RP and STGD. Similarly, Hanumunthada et al. found no significant difference in CVI when compared between PR and STGD [28]. This finding may confirm that choroidal changes are secondary to RPE changes in patients with IRD rather than a primary alteration. The more prominent changes in LCA and CRD patients when compared to Usher syndrome and healthy controls could be attributed to the earlier onset of the disease in LCA and CRD and also the severe involvement of photoreceptors.

The current study has some limitations including the small sample size in some groups such as LCA and STGD subtypes. However, this study has definite strengths such as comparison of IRD patients with age- and sex-matched control groups and also evaluation of different subgroups of IRD.

In conclusion, patients with IRD show changes in choroidal structure. These changes cause reduced choroidal vascularity index as a novel marker to investigate the choroid. In general, changes in choroidal luminal areas are more prominent than the stromal areas. More severe diseases with an earlier onset such as LCA and CRD may result in more prominent changes in the CVI.

### Electronic supplementary material

Below is the link to the electronic supplementary material.

Supplementary Material 1

## Data Availability

The datasets used and/or analyzed during the current study are available from the corresponding author on reasonable request.
