# Supplementary material for: Choroidal structure investigated by choroidal vascularity index in patients with inherited retinal diseases
Source: Int J Retina Vitreous. 2023 Mar 28;9:18. doi: 10.1186/s40942-023-00457-w (PMC10044756; doi:10.1186/s40942-023-00457-w)
Supplement: Supplementary file 1 — Supplementary Material 1 [file 40942_2023_457_MOESM1_ESM.docx]

**Reviewer reports:**

Reviewer #1: Dear authors,
Your article "Choroidal Structure Investigated by Choroidal Vascularity Index in Patients with Inherited Retinal Diseases" brings an interesting theme. It is well written.
Few questions/suggestions:
**1. Background is too long. Make it shorter.**

**Authors’ response:** Thanks for the comment. The introduction has been shortened according to the reviewer’s comment.

2. As we know choroidal thickness changes accordingly to its region. On IRD eyes, retina's anatomy is not well defined. In some cases, it is hard to identify the center of the macula. Based on that, how do you know you had compared the same location? Aren't you comparing the areas of different locations on different eyes? That could change your results.


**Authors’ response:** Thanks for the comment. The images with poor quality were excluded comprising the images in which the macula could not be identified at all. We clarified this issue in the method section.

Reviewer #2: The authors study the choroidal structure of 113 eyes of 113 patients with genetic testing proved inherited retinal diseases compared with the same number of controls. The method is well explained. This may be welcome to put light in the site where the clinical sings begin. 
The comments are on the attached file.
**Authors’ response:** Thanks for the comments.

Reviewer #3: Dear Dr Bayat,

Congratulations for your work. Different OCT biomarkers have been described before, including biomarkers of the choroidal vasculature.

I would like to make some comments:

**1-) It would be nice to see the genetic testing results, or at least you could describe them in the results.**

**Authors’ response:** We would like to thank the reviewer for the thoughtful comment. As we have referenced in the methods section of the manuscript “Patients underwent genetic testing to confirm the diagnosis. 23“ the patients have been selected from the national inherited retinal disease registry in Iran and in that article (reference 23), we have presented the results of genetic screening. We are also preparing another manuscript about the genetic status of Iranian IRD patients from a larger cohort. The reported patients in this manuscript are a fraction of those patients. Therefore, we prefer not to report the genetic data in this manuscript especially because the emphasis of this manuscript is mainly on clinical findings. We just wanted to indicate that the reported patients had genetically confirmed diagnosis. In addition, we inserted a paragraph in the results section to show that we did not find any association between the causative genes and the choroidal structures “The patients were very genetically heterogenous and we did not find any association between the causative genes and the choroidal structure (data not shown).”

2-) Some minor English typos. For example, in the Table 2: CVI: Choroidal Vascularity Index; CRD: Choroidal Vascularity Index

**Authors’ response:** Thanks for the comment. The typos have been corrected.
